# Management of pain in newborn circumcision: a systematic review

**DOI:** 10.1007/s00431-020-03758-6

**Published:** 2020-08-03

**Authors:** Serena Rossi, Giuseppe Buonocore, Carlo Valerio Bellieni

**Affiliations:** Department of Molecular and Developmental Medicine, University of Siena, Siena, Italy

**Keywords:** Male circumcision, Newborn pain, Analgesia, Sensorial saturation

## Abstract

Male circumcision (MC) is one of the most common surgical procedures performed on neonates. In the last decades, there have been consistent advances in the understanding of pain mechanisms in newborns, and analgesia has become a fundamental part of neonatal care. MC is still often performed with inappropriate analgesic methods, and there is still great variability among the various centers about surgical and anesthethic techniques to do it. The purpose of this review is to summarize the findings in the literature about pain management and analgesia during newborn MC. We performed a systematic review of neonatal MC studies published in the last 20 years. The most effective technique appeared to be the combination of pharmacological and non-pharmacological methods of analgesia.

*Conclusion*: Combining local anesthesia with non-pharmacological analgesic strategies appears to be effective preventing procedural pain during MC. However, a standardized protocol for analgesia during MC is yet to be determined. Sensorial saturation appeared to help when used in conjunction with the local anesthesia techniques.

**Table:** 

**What is Known:** *• Male circumcision is a painful procedure and it is frequently performed with inappropriate analgesic methods.* *• A gold standard practice in analgesia during male circumcision is still lacking and there is a great variability in the modus operandi between centers.*	
**What is New:** *• The combination of RB + EMLA + sucrose appears to be an analgesic strategy superior to other approaches.* *• We advocate for the integration of sensorial saturation during male circumcision in order to improve the efficacy of current analgesic practices.*

**What is Known:**

*• Male circumcision is a painful procedure and it is frequently performed with inappropriate analgesic methods.*

*• A gold standard practice in analgesia during male circumcision is still lacking and there is a great variability in the modus operandi between centers.*

**What is New:**

*• The combination of RB + EMLA + sucrose appears to be an analgesic strategy superior to other approaches.*

*• We advocate for the integration of sensorial saturation during male circumcision in order to improve the efficacy of current analgesic practices.*

## Introduction

Male circumcision (MC) is one of the oldest and most common surgical procedures in the world. It consists of the shaft skin and inner foreskin surgical removal to uncover the glans. The global prevalence of MC is estimated to be 38–39% [1]. Given the high rates of circumcision among Muslim and Jewish males, the prevalence of this procedure in the Middle East and North Africa reaches over 95%. In the USA, the prevalence of MC is 91% in White males, 76% in Black males, and 44% in Hispanic males [2]. In other English-speaking countries, the prevalence of MC is about 20–30%. In Asian and European countries, the prevalence ranges broadly and this is mainly dependent on the predominant religious beliefs [1]. MC can be performed at any age; it is most commonly performed in neonates, followed by infants and children with important differences in complication rates and related costs [3, 4].

Neonatal MC is a simple procedure, healing within 1 week, and when performed in a hospital setting by trained physicians, it has a low rate of adverse events [5]. The reasons for MC vary but most newborns are circumcised for religious or cultural reasons. There is considerable evidence of the health benefits connected to newborn male circumcision in preventing infectious and non-infectious diseases (i.e., prevention of urinary tract infections, acquisition of HIV, balanitis, paraphimosis, candidiasis, transmission of some sexually transmitted infections, and genital cancer) [5–7]. By contrast, some are less likely to endorse this procedure, and a debate is still ongoing in the medical community whether to recommend neonatal circumcision.

Three surgical devices are commonly used to perform MC: the Gomco clamp, the Plastibell device, and the Mogen clamp (Table 1).

**Table 1:** Surgical procedures for male circumcision

Procedure	Description
Gomco clamp	The device is placed over the glans; the foreskin is pulled over the bell. Gomco clamp is tightened and left in place a few minutes to allow for hemostasis. The foreskin is then cut with a scalpel [5].
Mogen clamp	It is a device with a slit-like space of 3 mm between two blades. The clamp is placed over the glans and the prepuce is positioned into the slit The blades are locked together, crushing the skin and creating hemostasis. The skin is excised from above the clamp [5].
Plastibell device	A plastic ring is applied and tied on to the foreskin at the level of desired foreskin removal. Tissue hemostasis is achieved. Removal of foreskin distal to the ligature [5].

MC performed without anesthesia is likely to be associated with intraoperative and post-operative pain. Therefore, MC should always be performed using anesthesia and post-operative analgesia [5, 8]. The relief of pain during medical procedures is a crucial goal for healthcare providers, and strong evidence shows that newborns can experience pain and that neuronal pathways are affected by painful stimuli as well as the future pain threshold [9, 10]. For the risk of neurological damage secondary to the use of general anesthetics, general anesthesia for MC in newborns should be avoided [11]. The use of local anesthesia for MC has proven to be a safe and effective way to prevent procedural pain both in neonates and in older children [5, 12–15].

For many years, physicians have tried to search for analgesic methods to perform MC safely but a gold standard for this practice has not yet been found. The aim of the present study is to systematically review the studies on MC pain management published during the past two decades.

## Methods

**Fig. 1:**
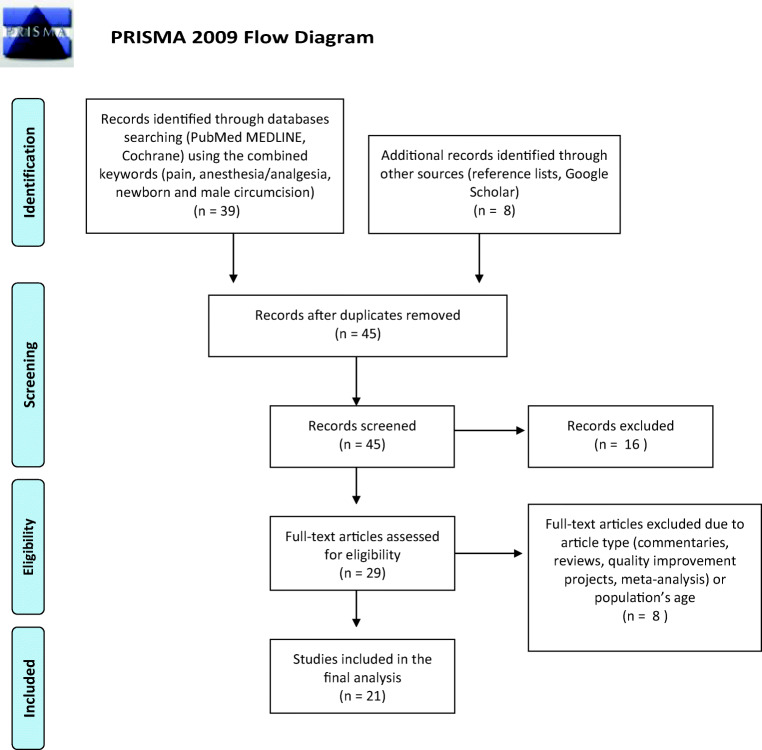
PRISMA 2009 flow diagram

We included in our search the original studies, published in the last two decades, comparing the effects of different types of anesthesia/analgesia for neonatal MC or the effects of different surgical techniques for MC on pain levels. We included only studies in which pain level was assessed using specific pain scales or physiologic and behavioral responses to pain. We considered only the studies in which MC was performed during the neonatal period. Only studies written in English were included in the analysis. Reviews, meta-analyses, commentaries, and quality improvement projects were excluded. We retrieved papers from PubMed, MEDLINE, and Cochrane databases using the following combining keywords and MeSH terms: pain, anesthesia, analgesia, newborn, and male circumcision. We consensually decided to use the aforementioned keywords altogether, aiming to increase the possibility of retrieving well-matched studies. Additional sources used to identify studies included the reference lists of relevant articles and Google Scholar. The final literature search was performed in January 2020 initially with the scanning of titles and abstracts for inclusion and then with the assessment of full-text articles performed by two authors independently (CVB and SR) in order to reduce the possibility of rejecting relevant reports. The study selection process is displayed using the PRISMA flow diagram [16] (Fig. 1). Data were extracted by one author (SR) using a data extraction form, which was independently checked by a second researcher (CVB). Data extracted included the year of publication, the study type, the sample size, the analgesic methods applied, the pain scale used, the surgical technique utilized, and the main results of each study. The data collected were summarized descriptively.

## Results

Forty-seven papers published from the year 2000 were retrieved after initial database search using the aforementioned combined keywords. Forty-five were screened after duplicate removal. Sixteen of them were excluded after the initial scanning of titles and abstract, and eight were excluded after full-text analysis because they did not fulfill our inclusion criteria. Twenty-one studies met the inclusion criteria and were included in the analysis.

Seventeen articles compared the effect of different analgesic methods in newborns’ MC [17–33] while 4 articles compared the effects of different surgical techniques on pain levels [35–38]. Thirteen studies enrolled more than 20 patients for each study group [17, 18, 20, 21, 23, 24, 27–33].

Table 2 displays, in chronological order, a summary of the key features and findings of each of the articles that met the inclusion criteria. Table 3 shows the articles comparing different surgical techniques.

**Table 2:** Comparison of analgesic methods for male circumcision

Authors	Sample size (*n*)	Pain scale	Technique	Results
Kass et al. [17]	47	crying time/MBPS/heart rate/respiratory rate	Gomco	*DPNB* (*n* = 24) vs. dextrose solution (*n* = 23)
Macke et al. [18]	60	NCAFS/crying time/heart rate	Gomco	Acetaminophen vs. placebo
Malnory et al. [20]	53	NIPS	Gomco	*Acetaminophen* vs. placebo
Choi et al. [21]	63	NIPS	unknown	EMLA and sodium chloride solution DPNB (*n* = 31) vs. placebo cream and bupivacaine DPNB (*n* = 32)
Lehr et al. [23]	53	heart rate/respiratory rate/SpO2	Gomco	DPNB vs. EMLA vs. Lidocaine 4% cream
South et al. [24]	44	PIPP/heart rate/crying time	Gomco	DPNB + Tylenol (*n* = 22) vs. *DPNB + Tylenol + non-nutritive sucking* (*n* = 22)
Lehr et al. [27]	53	NFCS/crying time	Gomco	DPNB vs. EMLA vs. Lidocaine 4% cream
Banieghbal et al. [28]	581	NIPS	Gomco	RB vs. *RB + milk/sucrose*
Bilgen et al. [29]	50	NIPS	Unknown	Caudal block with low volume high concentration bupivacaine vs. Caudal block with standard dose bupivacaine
Roman-Rodriguezet al. [30]	791	NIPS	Gomco	sucrose vs. *RB + sucrose*
Al Qahtani et al. [31]	90	N-PASS	Plastibell	EMLA vs. sucrose vs. *EMLA + sucrose*
Sharara-Chami et al. [32]	70	NIPS	Gomco	EMLA vs. EMLA + sucrose vs. EMLA + sucrose + DPNB vs. *EMLA + sucrose + RB*
Modekwe et al. [33]	110	SpO2	Plastibell	EMLA vs. *DPNB*

In italics are the most effective analgesic methods and the studies using validated pain scales

**Table 3:** Comparison of surgical techniques for male circumcision

Authors	Sample size	Technique	Analgesia
Taddio et al. [36]	86	*Mogen* vs. Gomco	DPNB + EMLA + acetaminophen (Mogen)/EMLA (Gomco)
Kaufman et al. [37]	57	*Mogen* vs. Gomco	EMLA + sucrose /EMLA + water DPNB + sucrose
Taeusch et al. [38]	59	Mogen vs. Plastibell	DPNB + sucrose
Sinkey et al. [39]	274	*Mogen* vs. Gomco	RB + sucrose

The main pharmacological methods used in the retrieved trials were as follows: EMLA cream which is an eutectic mixture of local anesthetics, with 2.5% lidocaine and 2.5% prilocaine producing dermal analgesia when applied as a topical cream on the skin 60–90 minutes before the procedure [5, 22, 26, 31, 32]; dorsal penile nerve block (DPNB) which involves regional anesthesia often obtained with 0.4 ml of 1% lidocaine or with bupivacaine injected at the base of the penis at the 10:00 and 2:00 positions [5, 17, 21, 23–25]; subcutaneous penile ring block (RB) which consists of the injection of 0.8 ml of 1% lidocaine in a circumferential ring around either the midshaft or at the level of the corona [5, 30, 32].

Other pharmacological interventions used were caudal block with bupivacaine [29], topical lidocaine cream (LMX4) containing 4% lidocaine [23], and acetaminophen/tylenol administered at the dosage of 10 mg/kg [18, 20, 24]. Among the non-pharmacological measures for pain control, the most commonly used were breast milk ad libitum during the procedure [28], sucrose solution (24–25% sucrose) [22, 30–32], dextrose solution (50% dextrose) [17], non-nutritive sucking (NNS) provided in the form of a gloved finger [24], and audio-stimulation with music (lullabies or repetitive rhythms) [19].

The ways used to assess pain response in neonates were both specific pain scales and physiological and behavioral responses to pain. NIPS (Neonatal Infant Pain Scale) was the most commonly used. Other methods to establish pain level were PIPP (premature infant pain profile), FLACC (Face, Legs, Activity, Cry, Consolability scale), NFCS (Neonatal Facial Coding System), N-PASS (Neonatal Pain, Agitation, and Sedation Scale), MBPS (Modified Behavioral Pain Scale), crying time, and the assessment of vital signs such as heart rate, oxygen saturation, and respiratory rate changes.

Ten articles, with more than 20 patients enrolled for each group, recorded pain levels during MC using validated pain scales [18, 20, 21, 24, 27–32].

### Dorsal penile nerve block

Ten papers compared DPNB with other anesthetic techniques [17, 21–27, 32, 33].

In two papers, DPNB was performed using bupivacaine [21, 25]; in one, DPNB with bupivacaine was proven more effective than DPNB with lidocaine [25]. DPNB with lidocaine was used for all the other studies [17, 22–24, 26, 27, 32, 33]. In 6 studies, DPNB was found to be more effective than the alternative measures used [17, 21, 22, 26, 32, 33]. In 4 studies, DPNB was combined with other forms of analgesia such as sucrose, EMLA, tylenol, and non-nutritive sucking. In all, the combined use of DPNB and additional pain-relieving methods proved to be effective [22, 24, 25, 32].

### EMLA

Nine studies focused on the effects of EMLA topical cream in producing analgesia during MC comparing it with other methods [19, 21–23, 26, 27, 31–33]. One study showed higher pain scores when EMLA cream was used as a sole analgesic method [32], and in the papers by Garry et al. and by Modekewe et al., EMLA was proven inferior to DPNB [26, 33]. Two studies demonstrated that the effectiveness of EMLA was higher when combined with other analgesic strategies, either pharmacological or non-pharmacological [31, 32]. Three studies found no significant differences in the comparison of EMLA with other analgesic strategies, namely DPNB and lidocaine cream [21, 23, 27]. In one study, EMLA proved to be more effective when combined with music [19]. An older study by Russell et al., in which MC was performer with Plastibell technique, reported a complete alleviation of pain with the application of EMLA 1 h prior to the procedure [34]. Another study by Taddio et al. in 1997 recorded behavioral (facial activity and time spent crying) and physiologic (heart rate and blood pressure) responses during MC performed with Gomco clamp, and demonstrated that EMLA is efficacious and safe for the prevention of pain from circumcision in neonates [35].

### Ring block

Four papers analyzed the effectiveness of ring block anesthesia either alone or combined with other strategies [22, 28, 30, 32]. RB combined with oral sucrose was found to be more effective than RB alone [22, 30]. In one study, RB combined with EMLA and sucrose was significantly more effective than both EMLA + sucrose and DPNB + EMLA + sucrose [32]. In one study, RB along with breast milk and/or 20% sucrose resulted in painless circumcision for neonates under 2 weeks old, with a NIPS score of 0 to 2 [28].

### Caudal block

One study assessed the effectiveness of caudal block performed either with low-volume high-concentration bupivacaine (0.375%) or standard dose of bupivacaine (0.25%). Block onset time and NIPS score were identical between the two groups demonstrating the effectiveness of high-concentration bupivacaine [29].

### Acetaminophen

Two studies compared the use of acetaminophen vs. placebo to obtain analgesia during MC [18, 20]. One study found no difference between the two groups [18]; by contrast, the paper by Malnory et al. showed a stronger analgesia in newborns receiving acetaminophen [20].

### Lidocaine cream

Two papers evaluated the use of lidocaine 4% cream comparing it with lidocaine 2.5% cream, EMLA, and DPNB. Pain response was evaluated using the changes in vital signs and no significant modifications in heart rate were found with different analgesic methods. Mean respiratory rate during the procedure was lower with lidocaine 4% cream and with DPNB compared with EMLA [23, 27].

### Sucrose

Results from multiple studies discourage the use of sucrose alone due to the lack of its analgesic effect [17, 22, 30, 31].

## MC surgical techniques

Thirteen out of 17 studies disclosed which surgical techniques were used for MC, Gomco being the most commonly utilized. Four papers compared the differences in pain profile with the use of different surgical techniques [36–39]. All studies demonstrated that MC performed with Mogen clamp was faster. Three papers compared Mogen clamp with Gomco clamp, demonstrating that Mogen clamp causes less pain especially when combined with preoperative analgesia (either with DPNB plus EMLA and acetaminophen, RB plus sucrose, or EMLA plus sucrose) [36, 37, 39]. Another study compared Mogen clamp with Plastibell procedure, using DPNB + dextrose as analgesia, finding no significant differences in terms of crying time [38].

## Discussion

It was once thought that newborns were unable to feel pain and that painful sensations experienced during the neonatal period would have easily been forgotten by them. It is now known, however, that newborns do feel pain and there is evidence that this can have physiological and behavioral consequences. Newborns are strongly susceptible to painful stimuli because, while ascending neural pathways for nociception are well-developed by the second trimester of pregnancy, the modulatory descending pathways are immature, even in term newborns [40–42]. There is ongoing research regarding the effects of prolonged or repetitive painful stimuli on newborns’ neurodevelopment, provoking a decreased brain growth in the frontal and parietal lobes and alterations in organization of neuronal connections in the temporal lobes [9, 43, 44]. Taddio et al. demonstrated a stronger pain response to routine vaccination in infants circumcised without anesthesia in comparison with uncircumcised infants or circumcised babies treated with topical anesthesia; they argued that MC without anesthetic may induce changes in infant pain behavior because of alterations in the neural processing of painful stimuli [10]. By contrast, a recent analysis by O’Callahan et al. demonstrated that newborns circumcised with proper analgesia (24%sucrose plus local anesthesia with lidocaine or DPNB and acetaminophen) showed no differences in the rate of exclusive breastfeeding during the initial hospitalization compared with non-circumcised newborns [45]. Therefore, it is imperative for clinicians to attempt all the possible strategies to reduce neonatal pain, and since MC is an invasive painful procedure, performing it without anesthesia appears to be contraindicated [8].

The studies we retrieved displayed heterogeneity in terms of sample size, pain scales, analgesic methods, and surgical techniques used. In some studies, the surgical details were not specified, and others used non-specific methods for pain evaluation (e.g., crying time, heart rate, respiratory rate). As a consequence and limitation of the study, it was not possible to perform a meta-analysis of different studies. Analyzing the studies with more than 20 patients for each study group and that used a validated pain scale, it was clear that the best results were obtained with pharmacologic local anesthesia and in particular the combination of pharmacological and non-pharmacological methods [20, 21, 24, 27–32]. As demonstrated by South et al., adding non-nutritive sucking to a combination of DPNB and tylenol resulted in lower pain scores compared with the use of pharmacological methods alone [24].

The value of combining non-pharmacological analgesic methods with DPNB has been demonstrated not only in newborns but also in older infants [12]. Similarly, by adding sucrose to other analgesic methods, either EMLA, DPRB, or RB, the result was a minimal pain level during MC [30–32]. These findings are in accordance with the work by Stevens at al. demonstrating the value of sucrose administration during painful procedures in neonates [46].

Only one study compared DPNB with RB, both associated with EMLA and sucrose, demonstrating a better analgesic effect when the combination of RB + EMLA + sucrose was used [32]. This finding confirmed the results of an older study by Lander et al. showing that RB is superior to DPNB [47].

Regarding surgical techniques, the general belief is that Mogen technique is shorter in terms of duration compared with Gomco or Plastibell [36–39]. A large study by Sinkey et al. suggests that Mogen technique is also less painful than Gomco [39]. However, in the majority of trials comparing analgesic methods that were considered in this review, MC was performed using Gomco. One reason for this might be the fact that the choice of the surgical technique is variable between centers, and there seems to be a trend toward the use of Gomco in Western countries’ in-hospital settings [48, 49].

Two studies focused mainly on post-operative analgesia showing lower pain levels and longer-lasting analgesia with the use of bupivacaine as an anesthetic agent during MC [25, 29].

Despite the great variability that undoubtedly exists regarding the provision of analgesia during MC, the use of a combined pharmacological and non-pharmacological approach could be considered safe and effective. As we stated in our previous work on this topic, we are convinced that a possible valuable adjunct to the current perspective might be the integration of sensorial saturation (SS) as an aid to pharmacological analgesia for MC [50]. SS is a validated method to reduce newborns’ stress response to nociception and is based on the so-called gate-control theory by which the brainstem can filter and reduce the transmission of pain to the brain if the person is concentrated on something else. In this way, by attracting babies’ attention with positive stimuli (tactile, auditory, gustatory, and visual), it is possible to reduce and even nullify the perception of painful stimuli [51].

In conclusion, since a standardized protocol for analgesia during MC is yet to be determined, we encourage more research in this field as a possible implementation of current clinical practice, in order to ameliorate the current strategies so that newborns’ MC might eventually become a painless procedure. Given the proven effects of SS, a possible strategic direction to pursue in the field of MC analgesia could be the combination of pharmacological methods with SS, possibly potentiating the already proven positive effects of sucrose in conjunction with anesthetic drugs.

## Abbreviations

DPNB: Dorsal penile nerve block
EMLA: Eutectic mixture of local anesthetics
FLACC: Face, Legs, Activity, Cry, Consolability scale
LMX4: Lidocaine 4% cream
MBPS: Modified Behavioral Pain Scale
MC: Male circumcision
NFCS: Neonatal Facial Coding System
NIPS: Neonatal Infant Pain Scale
NNS: Non-nutritive sucking
N-PASS: Neonatal Pain, Agitation, and Sedation Scale
PIPP: Premature infant pain profile
RB: Ring block
SS: Sensorial saturation

## References

[1] Morris BJ, Wamai RG, Henebeng EB, Tobian AA, Klausner JD, Banerjee J, Hankins CA (2016). Estimation of country-specific and global prevalence of male circumcision. Popul Health Metrics.

[2] Introcaso CE, Xu F, Kilmarx PH, Zaidi A, Markowitz LE (2013). Prevalence of circumcision among men and boys aged 14 to 59 years in the United States, National Health and Nutrition Examination Surveys 2005-2010. Sex Transm Dis.

[3] Joint United Nations Programme on HIV/AIDS (UNAIDS). Neonatal and child male circumcision: a global review. April, 2010. Available at: https://www.who.int/hiv/pub/malecircumcision/neonatal_child_MC_UNAIDS.pdf

[4] Many BT, Rizeq YK, Vacek J, Cheon EC, Johnson E, Hu YY, Raval MV, Abdullah F, Goldstein SD (2020). A contemporary snapshot of circumcision in US children’s hospitals. J Pediatr Surg.

[5] Blank S, Brady M, Buerk E, Carlo W, Diekema D, Freedman A, Maxwell L, Wegner S (2012). Male circumcision. Pediatrics.

[6] Simpson E, Carstensen J, Murphy P (2014). Neonatal circumcision: new recommendations & implications for practice. Mo Med.

[7] Morris BJ, Kennedy SE, Wodak AD, Mindel A, Golovsky D, Schrieber L, Lumbers ER, Handelsman DJ, Ziegler JB (2017). Early infant male circumcision: systematic review, risk-benefit analysis, and progress in policy. World J Clin Pediatr.

[8] Paix BR, Peterson S (2012). Circumcision of neonates and children without appropriate anaesthesia is unacceptable practice - reply. Anaesth Intensive Care.

[9] Walker SM (2019). Long-term effects of neonatal pain. Semin Fetal Neonatal Med.

[10] Taddio A, Katz J, Ilersich AL, Koren G (1997). Effect of neonatal circumcision on pain response during subsequent routine vaccination. Lancet.

[11] McPherson C, Inder T (2017). Perinatal and neonatal use of sedation and analgesia. Semin Fetal Neonatal Med.

[12] Nguyen TT, Kraft E, Nasrawi Z, Joshi M, Merianos D (2019). Avoidance of general anesthesia for circumcision in infants under 6 months of age using a modified Plastibell technique. Pediatr Surg Int.

[13] Brady-Fryer B, Wiebe N, Lander JA (2004) Pain relief for neonatal circumcision. Cochrane Database Syst Rev (3) Art. No.: CD004217. 10.1002/14651858.CD004217.pub2 10.1002/14651858.CD004217.pub2 PMC8768484 15495086

[14] Sandeman DJ, Dilley AV (2007). Ultrasound guided dorsal penile nerve block in children. Anaesth Intensive Care.

[15] Sandeman DJ, Reiner D, Dilley AV, Bennett MH, Kelly KJ (2010). A retrospective audit of three different regional anaesthetic techniques for circumcision in children. Anaesth Intensive Care.

[16] Moher D, Liberati A, Tetzlaff J, Altman DG, The PRISMA Group (2009). Preferred reporting items for systematic reviews and meta-analyses: the PRISMA statement. PLoS Med.

[17] Kass FC, Holman JR (2001). Oral glucose solution for analgesia in infant circumcision. J Fam Pract.

[18] Macke JK (2001). Analgesia for circumcision: effects on newborn behavior and mother/infant interaction. J Obstet Gynecol Neonatal Nurs.

[19] Joyce BA, Keck JF, Gerkensmeyer J (2001). Evaluation of pain management interventions for neonatal circumcision pain. J Pediatr Health Care.

[20] Malnory M, Johnson TS, Kirby RS (2003). Newborn behavioral and physiological responses to circumcision. MCN Am J Matern Child Nurs.

[21] Choi WY, Irwin MG, Hui TWC, Lim HH, Chan KL (2003). EMLA cream versus dorsal penile nerve block for postcircumcision analgesia in children. Anesth Analg.

[22] Razmus S, Dalton ME, Wilson D (2004). Pain management for newborn circumcision. Pediatr Nurs.

[23] Lehr VT, Cepeda E, Frattarelli DAC, Thomas R, LaMothe J, Aranda JV (2005). Lidocaine 4% cream compared with lidocaine 2.5% and prilocaine 2.5% or dorsal penile block for circumcision. Am J Perinatol.

[24] South MMT, Strauss RA, South AP, Boggess JF, Thorp JM (2005). The use of non- nutritive sucking to decrease the physiologic pain response during neonatal circumcision: a randomized controlled trial. Am J Obst Gynecol.

[25] Stolik-Dollberg OC, Dollberg S (2005). Bupivacaine versus lidocaine analgesia for neonatal circumcision. BMC Pediatr.

[26] Garry DJ, Swoboda E, Elimian A, Figueroa R (2006). A video study of pain relief during newborn male circumcision. J Perinatol.

[27] Lehr VT, Zeskind PS, Ofenstein JP, Cepeda E, Warrier I, Aranda JV (2007). Neonatal facial coding system scores and spectral characteristics of infant crying during newborn circumcision. Clin J Pain.

[28] Banieghbal B (2009). Optimal time for neonatal circumcision: an observation-based study. Pediatr Urol.

[29] Bilgen S, Koner O, Menda F, Karacay S, Kaspar EC, Sozubir S (2013). A comparison of two different doses of bupivacaine in caudal anesthesia for neonatal circumcision. A randomized clinical trial. Middle East J Anaesthesiol.

[30] Roman-Rodriguez CF, Toussaint T, Sherlock DJ, Fogel J, Hsu CD (2014). Pre-emptive penile ring block with sucrose analgesia reduces pain response to neonatal circumcision. Urology.

[31] Al Qahtani R, Abu-Salem L, Pal K (2014). Effect of lidocaine-prilocaine eutectic mixture of local anaesthetic cream compared with oral sucrose or both in alleviating pain in neonatal circumcision procedure. Afr J Paediatr Surg.

[32] Sharara-Chami R, Lakissian Z, Charafeddine L, Milad N, El-Hout Y (2017). Combination analgesia for neonatal circumcision: a randomized controlled trial. Pediatrics.

[33] Modekwe VI, Ugwu JO, Ekwunife OH, Osuigwe AN, Obiechina SO, Okpalike IV, Orakwe JC (2019). Comparison of the efficacy of eutectic mixture of local anesthetics (EMLA) and dorsal penile nerve block (DPNB) in neonatal circumcision. Niger J Clin Pract.

[34] Russell CT, Chaseling J (1996). Topical anaesthesia in neonatal circumcision: a study of 208 consecutive cases. Aust Fam Phys.

[35] Taddio A, Stevens B, Craig K, Rastogi P, Ben-David S, Shennan A, Mulligan P, Koren G (1997). Efficacy and safety of lidocaine–prilocaine cream for pain during circumcision. N Engl J Med.

[36] Taddio A, Pollock N, Gilbert-MacLeod C, Ohlsson K, Koren G (2000). Combined analgesia and local anesthesia to minimize pain during circumcision. Arch Pediatr Adolesc Med.

[37] Kaufman GE, Cimo S, Miller LW, Blass EM (2002). An evaluation of the effects of sucrose on neonatal pain with 2 commonly used circumcision methods. Am J Obstet Gynecol.

[38] Taeusch HW, Martinez AM, Partridge JC, Sniderman S, Armstrong-Wells J, Fuentes-Afflick E (2002). Pain during Mogen or PlastiBell circumcision. J Perinatol.

[39] Sinkey RG, Eschenbacher MA, Walsh PM, Doerger RG, Lambers DS, Sibai BM, Habli MA (2015). The GoMo study: a randomized clinical trial assessing neonatal pain with Gomco vs Mogen clamp circumcision. Am J Obstet Gynecol.

[40] Fitzgerald M (2015). What do we really know about newborn infant pain?. Exp Physiol.

[41] Fitzgerald M (2000) Development of pain pathways and mechanisms. In: McGrath PJ, Anand JKS, Stevens BJ (eds) Pain in neonates, 2nd edn. Elsevier, pp 9–21

[42] Lago P, Ancora G, Bellieni CV, Cavazza A, Cocchi G, Guadagni AM, Memo L, Merazzi D, Pirelli A (2006). Linee-guida e raccomandazioni per la prevenzione ed il trattamento del dolore nel neonato. Pediatr Med Chir.

[43] Walker SM (2014). Neonatal pain. Paediatr Anaesth.

[44] McPherson C, Grunau RE (2014). Neonatal pain control and neurologic effects of anesthetics and sedatives in preterm infants. Clin Perinatol.

[45] O’Callahan C, Te S, Husain A, Rosener SE, Hussain N (2020). The effect of circumcision on exclusive breastfeeding, phototherapy, and hospital length of stay in term breastfed newborns. Hosp Pediatr.

[46] Stevens B, Yamada J, Ohlsson A, Haliburton S, Shorkey A (2016) Sucrose for analgesia in newborn infants undergoing painful procedures. Cochrane Database Syst Rev 7(7). 10.1002/14651858.CD001069.pub5 10.1002/14651858.CD001069.pub5 PMC6457867 27420164

[47] Lander J, Brady-Fryer B, Metcalfe JB, Nazarali S, Muttitt S (1997). Comparison of ring block, dorsal penile nerve block, and topical anesthesia for neonatal circumcision: a randomized controlled trial. JAMA..

[48] Chan PS, Penna FJ, Holmes AV (2018). Gomco versus Mogen? No effect on circumcision revision rates. Hosp Pediatr.

[49] Heras A, Vallejo V, Pineda MI, Jacobs AJ, Cohen L (2018). Immediate complications of elective newborn circumcision. Hosp Pediatr.

[50] Bellieni CV, Alagna MG, Buonocore G (2013). Analgesia for infants’ circumcision. Ital J Pediatr.

[51] Locatelli C, Bellieni CV (2018). Sensorial saturation and neonatal pain: a review. J Mater Fetal Neonatal Med.

